# Escape From X-Chromosome Inactivation: An Evolutionary Perspective

**DOI:** 10.3389/fcell.2019.00241

**Published:** 2019-10-22

**Authors:** Bronwyn J. Posynick, Carolyn J. Brown

**Affiliations:** Department of Medical Genetics, Molecular Epigenetics Group, Life Sciences Institute, The University of British Columbia, Vancouver, BC, Canada

**Keywords:** dosage compensation, X-chromosome inactivation, mammalian evolution, escape from X-chromosome inactivation, sex chromosomes, gametologues

## Abstract

Sex chromosomes originate as a pair of homologus autosomes that then follow a general pattern of divergence. This is evident in mammalian sex chromosomes, which have undergone stepwise recombination suppression events that left footprints of evolutionary strata on the X chromosome. The loss of genes on the Y chromosome led to Ohno’s hypothesis of dosage equivalence between XY males and XX females, which is achieved through X-chromosome inactivation (XCI). This process transcriptionally silences all but one X chromosome in each female cell, although 15–30% of human X-linked genes still escape inactivation. There are multiple evolutionary pathways that may lead to a gene escaping XCI, including remaining Y chromosome homology, or female advantage to escape. The conservation of some escape genes across multiple species and the ability of the mouse inactive X to recapitulate human escape status both suggest that escape from XCI is controlled by conserved processes. Evolutionary pressures to minimize dosage imbalances have led to the accumulation of genetic elements that favor either silencing or escape; lack of dosage sensitivity might also allow for the escape of flanking genes near another escapee, if a boundary element is not present between them. Delineation of the elements involved in escape is progressing, but mechanistic understanding of how they interact to allow escape from XCI is still lacking. Although increasingly well-studied in humans and mice, non-trivial challenges to studying escape have impeded progress in other species. Mouse models that can dissect the role of the sex chromosomes distinct from sex of the organism reveal an important contribution for escape genes to multiple diseases. In humans, with their elevated number of escape genes, the phenotypic consequences of sex chromosome aneuplodies and sexual dimorphism in disease both highlight the importance of escape genes.

## Introduction

**FIGURE 1:**
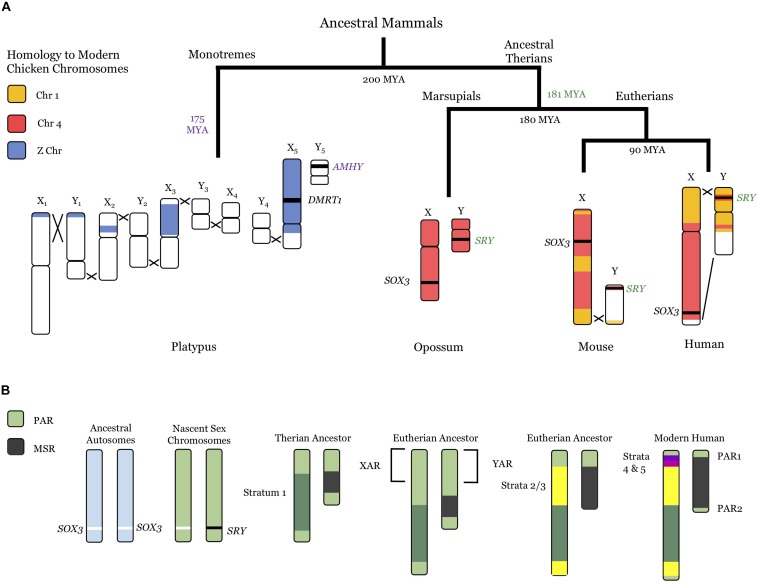
Evolution of mammalian sex chromosomes. **(A)** Current sex chromosomes of the platypus, opossum, mouse, and human, including recombination locations (black Xs) and homology to current chicken chromosomes (Z chromosome, blue; Chromosome 4, red; Chromosome 1, orange). Divergence times between lineages (black) and approximate dates of sex-determining mutations (monotremes, *AMHY*, purple text; marsupials and eutherians, *SRY*, green text) are also noted. There is variation in dates in the literature: those shown are from Cortez et al. (2014). See text for further references. **(B)** Evolutionary progression from autosomes (light blue, left) to modern human sex chromosomes (right). PARs, light green; Stratum 1, dark green; Strata 2/3, yellow; Stratum 4, fuchsia; Stratum 5, purple. The XAR/YAR regions from the ancestral chromosome 4 **(A)** are also noted. MYA, million years ago; PAR, pseudoautosomal region; MSR, male-specific region; XAR/YAR, X/Y added region.

Sex can be determined through various strategies, either environmentally or genetically. The evolution of chromosomal sex determination begins as the result of a sex-determining mutation on an autosome, favoring the loss of recombination at that location (reviewed in Bergero and Charlesworth, 2009; Bachtrog et al., 2014; Ponnikas et al., 2018). This nascent sex chromosome is then present in one sex (the heterogametic sex) but not the other (the homogametic sex), which retains the ancestral chromosome pair. Male heterogametic sex-determining systems (e.g., mammals), are termed XY, while female heterogametic systems (e.g., birds) are termed ZW. Y and W chromosomes are generally smaller and more heterochromatic, while X and Z chromosomes retain much of their ancestral gene complement (reviewed in Bachtrog et al., 2011). Progressive recombination suppression promotes further divergence between the sex chromosome pair: the Y or W chromosome rapidly degrades while the X or Z chromosome remains protected by recombination in the homogametic sex. This evolutionary divergence of the sex chromosomes has been well described in mammals (Figure 1).

Prototherians (monotremes) and therians (marsupials and placental mammals) appear to have developed their modern sex chromosome systems in tandem, following the divergence between the two lineages about 200 million years ago (MYA) (Cortez et al., 2014). Monotremes developed their current sex chromosomes roughly 175 MYA (Cortez et al., 2014): the male platypus has five X chromosomes (X_1–5_) and five Y chromosomes (Y_1–5_), while the female has two copies of each X_1–5_ (Grützner et al., 2004; Rens et al., 2007). Four of the platypus X chromosomes share homology with the bird Z chromosome (Veyrunes et al., 2008), with the bird sex-determining gene (*DMRT1*) present on X_5_ (Smith et al., 2009). The best candidate for the monotreme sex-determining gene is the anti-mullerian hormone (AMHY) gene on Y_5_ (Cortez et al., 2014), which is important in other vertebrate sex-determining pathways (Hattori et al., 2012). Notably, the therian sex-determining system is entirely divergent from monotremes, and likely arose just prior to the split between marsupials and placental mammals (Potrzebowski et al., 2008; Cortez et al., 2014).

Modern therian sex chromosomes originated from a pair of autosomes homologous to the chicken chromosome 4 (Lahn and Page, 1999; Ross et al., 2005). A *SOX3* mutation likely resulted in the sex-determining male gene (*SRY*), creating *de facto* sex chromosomes (Sinclair et al., 1990; Stevanovlć et al., 1993). Strong selection for linkage between *SRY* and other male-specific genes resulted in stepwise recombination suppression between the ancestral X and Y chromosomes and created strata of different divergence eras (Rice, 1984; Lahn and Page, 1999; Skaletsky et al., 2003; Ross et al., 2005; Bergero and Charlesworth, 2009). Throughout evolution, the male-specific region (MSR) present only on the Y chromosome expanded, while the X–Y homologous pseudoautosomal region (PAR) that is essential for proper pairing with the X chromosome diminished (reviewed in Bergero and Charlesworth, 2009). The first recombination suppression event appears to have been an inversion in the last common therian ancestor, shown as Stratum 1. In placental mammals, an autosomal region homologous to chicken chromosome 1 (X added region, XAR) was added prior to the placental radiation, apparently around the same time as Strata 2/3 were created through further recombination suppression (Spencer et al., 1991; Ross et al., 2005; Cortez et al., 2014). Interestingly, more sophisticated bioinformatic approaches have found evidence for seven distinct strata within Strata 1–3 (Pandey et al., 2013), indicative of numerous recombination-suppressing events within those more distant time frames. The XAR also underwent successive inversion events, resulting in Stratum 4 and 5 (Lemaitre et al., 2009), and leaving the modern human PAR (PAR1) (Lahn and Page, 1999; Ross et al., 2005). A second pseudoautosomal region, PAR2, is present in humans (Charchar et al., 2003).

As these recombination suppressing events occurred, the Y chromosome became progressively degraded, with the human only retaining about 3% of its ancestral genes (Skaletsky et al., 2003; Bellott et al., 2010). Although there has been speculation about an impending Y chromosome extinction (e.g., Aitken and Graves, 2002), the human Y has generally conserved its remaining genes over the last 25 million years (Hughes et al., 2012). Not surprisingly, many Y-linked genes are involved in male-specific processes such as spermatogenesis (Skaletsky et al., 2003); surviving X–Y pairs (gametologs) are largely involved in dosage-sensitive processes (Bellott et al., 2014), further discussed below.

Erosion of the Y chromosome leads to gene imbalance in males compared to the ancestral autosomal gene dosage. Susumu Ohno hypothesized that male mammals must upregulate their single X chromosome to match autosomal expression levels (Ohno, 1967), and that females balance dosage with males through X-chromosome inactivation (XCI, discussed below). While Ohno originally referred to XCI as a mechanism of dosage compensation, some research groups argue that dosage compensation occurs only in the heterogametic sex (Mank et al., 2011). Therefore, in this review we will refer to expression balance between the heterogametic sex chromosomes and the autosomes as dosage compensation, and that between male and female sex chromosomes as dosage equivalance.

There have been contradictory findings about dosage compensation in mice and humans, largely influenced by the filtering of genes examined (Castagné et al., 2011). Early microarray studies in mice supported Ohno’s hypothesis (Gupta et al., 2006; Nguyen and Disteche, 2006), but RNA-sequencing (RNA-seq) studies in mice and humans have been inconsistent (reviewed in Mank, 2013; Pessia et al., 2014; Disteche, 2016). RNA-seq studies that only analyzed highly expressed genes have supported Ohno’s hypothesis (Deng et al., 2011), although it has been noted that this filtering may enrich for dosage-sensitive genes, such as those in large protein complexes (see Pessia et al., 2012, for further discussion). Similarly, comparisons including testis-specific genes showed no evidence of global dosage compensation (Xiong et al., 2010), arguably since average transcription was decreased (Kharchenko et al., 2011). Notably, none of these studies considered the evolutionary age of the genes in question (He et al., 2011). Ohno’s hypothesis specifically refered to compensation between genes on the ancestral sex chromosome and their autosomal partners, and so ampliconic or elsewise added genes (Bellott et al., 2010) might not be expected to be dosage compensated. Additionally, particularly dosage-sensitive ancestral genes may have either been translocated to autosomes or duplicated into paralogous gene families (Hurst et al., 2015), obviating the need for dosage compensation. When RNA-seq data was compared between X-linked genes and their autosomal chicken homologs (Julien et al., 2012), there was no evidence for global X-upregulation. Intriguingly, evidence for downregulation of autosomal genes interacting with X-linked genes was observed, consistent with the need to maintain balance of expression between the sex chromosomes and the autosomes, as well as between the sexes.

## X-Chromosome Inactivation in Mammals

X-chromosome inactivation is an epigenetic process that occurs in the early development of therian females, transcriptionally silencing all but one of the X chromosomes present in each cell. First proposed in the 1960s (Lyon, 1961), silencing is initiated by the expression of a long non-coding RNA (lncRNA) on what will become the inactive X (Xi). The lncRNA spreads in *cis* to coat the future Xi, beginning a cascade of epigenetic changes that results in the formation of a dense heterochromatic Barr body (recently reviewed in Galupa and Heard, 2018). Once formed, the Xi is stably inherited through subsequent mitotic cell divisions. XCI is an imprinted process in marsupials (Richardson et al., 1971), mediated by expression of the lncRNA *Rsx* on the paternal X chromosome (Grant et al., 2012). In placental mammals, XCI is initiated by the lncRNA *XIST* (Brown et al., 1991), which likely evolved from the protein-coding gene *Lnx2b* (Duret et al., 2006). The region syntenic to the *XIST*-containing X inactivation center is rearranged in marsupials (Davidow et al., 2007) and *Lnx2b* remains protein-coding, suggesting that *XIST* arose after divergence between marsupials and placentals. The origin of *Rsx* is unknown; the syntenic region in eutheria is flanked by *HPRT* and *FAM122B*, and contains the placenta-specific gene *PLAC1*.

In placental mammals, XCI is random in embryonic tissues, although imprinted XCI occurs in the extraembryonic tissues of mice (Takagi and Sasaki, 1975). Therefore, females are usually mosaics of cells with either the paternal or the maternal active X chromosome (Xa). Random XCI requires the existence of a counting mechanism to ensure that one X chromosome is always active; this mechanism must distinguish between two functionally equivalent X chromosomes in the same nuclear environment. Although numerous models have been proposed, the factor(s) involved in this mechanism have not yet been identified (Loos et al., 2016; Migeon et al., 2017; Mutzel et al., 2019).

In the limited species examined to date, not all X-linked genes are silenced by XCI. Genes that are still expressed from the Xi are considered to “escape,” while genes that are silenced are “subject.” Some escape genes consistently escape inactivation, while others exhibit variable escape and are only expressed in some cell types or individuals. Conventionally, genes are considered to escape if they are expressed at a level that is at least 10% of the Xa allele (Carrel and Willard, 2005). The exclusively paternal marsupial XCI has 14–30% of X-linked genes escaping inactivation (Al Nadaf et al., 2011; Wang et al., 2014; Whitworth and Pask, 2016). Humans also have considerable expression from the Xi, with at least 15% consistently escaping and another 15% variably escaping XCI (Balaton et al., 2015). Mice appear to be more stringent in their silencing with only 3–7% of X-linked genes consistently escaping XCI, although more variable escape genes are being described (reviewed in Carrel and Brown, 2017). When discussing escape from XCI, it is generally unknown if escape occurs from reactivation or avoidance of an initial silencing signal.

## Genes that Escape From XCI

### An Evolutionary Perspective on Why Genes Escape XCI

**FIGURE 2:**
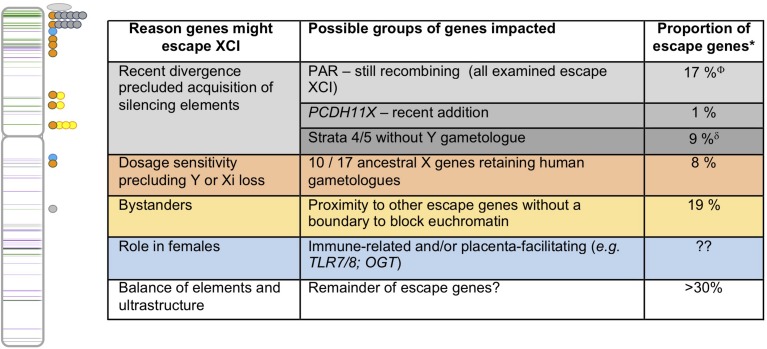
Human escape genes and possible underlying evolutionary reasons for their ongoing expression from the Xi. On the left is an ideogram of the X chromosome showing the genes that escape XCI (green) or variably escape XCI (purple) with lines appearing darker when multiple genes are nearby (data from Balaton et al., 2015). Circles to the right of the chromosome reflect locations of escape genes with features highlighted in the table to the right. The color of the dots matches the table, with PAR1 genes shown as an oval, given their abundance. ^∗^As tabulated in Balaton et al. (2015); ^Φ^Includes three ancestral X genes; ^δ^Includes four ancestral X genes.

There is no single model that satisfactorily explains the variability in both extent and specific genes that escape inactivation across species. There are likely multiple evolutionary paths that can result in ongoing expression from the Xi. Those X-linked genes that retain Y homology are argued to have critical dosage sensitivity that did not allow loss of expression from either the Y or the Xi (Bellott et al., 2014). However, not all genes that escape XCI need to be dosage-sensitive. Other X–Y gametologues with a more recent addition to the sex chromosomes, or a more recent divergence between them, may have neither lost Y expression nor gained X silencing capacity. Additionally, over half of the genes that escape from XCI lack functional Y gametologs (Balaton et al., 2015). Escape for these genes could simply reflect that the acquisition of features favoring silencing may not have occurred. If escape genes have DNA sequences that promote expression from the Xi then a flanking dosage-insensitive gene may also be expressed as a bystander effect. Additionally, selection might favor expression from the Xi in females. Below, and in Figure 2, we outline aspects of these scenarios, and conjecture which escape genes might be so explained.

#### Y Homology

The presence of Y homology is clearly a strong driver for escape, albeit for only a subset of genes. The most complete block of Y homology is the PAR, and in humans there is both a short arm PAR (PAR1) and a smaller long arm PAR (PAR2) (Figure 1). As initially proposed by Lyon, escape for these genes would allow dosage equivalence between males and females (Lyon, 1962). All of the genes examined to date within PAR1 do escape from XCI, although they generally show some male-bias in expression (Balaton et al., 2015; Tukiainen et al., 2017). Surprisingly, some PAR2 genes are silenced on both the Xi and the Y (Ciccodicola et al., 2000), highlighting another route to equalize expression between the sexes.

Outside of the PARs, ongoing Y homology still strongly correlates with escape from XCI, most strikingly for those genes that have retained functional Y gametologs (Carrel and Willard, 2005). In an analysis of eight mammals, convergent survival of 36 ancestral X–Y genes was observed (Bellott et al., 2014). The remarkable longevity of these pairs was argued to result from either acquisition of male-specific function, or the need to preserve dosage of critical regulatory genes. Indeed, many of the long-lived X-Y gametologous genes are involved in transcription/translation or chromatin modification, and 10 of the 17 ancestral gametologs in humans are known to escape XCI (Bellott et al., 2014; Balaton et al., 2015). These findings have been supported by a recent study that used the conservation of miRNA targeting to demonstrate that remaining X–Y pairs are more dosage sensitive than escape genes without gametologs (Naqvi et al., 2018).

#### Beyond Y Homology

As discussed above, the step-wise loss of recombination left footprints of evolutionary strata on the X chromosome. There is a clear correlation between these strata and the likelihood of escape from inactivation, with the more recently diverged genes being more likely to escape (Carrel and Willard, 2005). It has been suggested that elements that favor silencing accumulated on the X chromosome once it diverged from the Y chromosome. The potential nature of these elements will be discussed in section “Elements That Influence Escape From XCI.”

Selection could also favor escape from XCI if there is a female-specific benefit to ongoing expression from the Xi. Notably, the acquisition of an invasive placenta coincides evolutionarily with the addition of the XAR, which harbors an excess of escape genes. This change in reproductive biology required female eutherian immune systems to both be able to tolerate fetal antigens and defend against parasites and pathogens during pregnancy. The “pregnancy compensation hypothesis” recently proposed that sex-specific differences in both autoimmune diseases (female-biased) and most cancers (male-biased) in industrialized environments are due to adaptions that occured to faciliate successive pregnancies in ancestral conditions (Natri et al., 2019).

### Elements That Influence Escape From XCI

The progressive sequestering of recombination between the sex chromosomes likely coincided with different rates of acquisition for genetic elements that favor the spread of silencing. Escape genes may be more prevalent in the XAR due to a paucity of silencing elements in this region. Notably, escape genes are still expressed from older areas of the X chromosome and so must have also acquired genetic elements that still allow expression from the Xi. Domains of silencing and escape require boundaries between the different regions of closed versus open chromatin. Here we discuss the evidence for silencing, escape, and boundary elements, and the potential effects of the physical structure of the Xi. To date, these features have only been studied in humans and mice.

#### Silencing Elements

The autosomal portion of an unbalanced X;autosome translocation is able to establish stable silencing, although less efficiently than the X chromosome itself, which led to the proposal of “waystations” that propagate the silencing signal along the X chromosome (Gartler and Riggs, 1983). These waystations would need to be present in greater frequency on the X chromosome compared to the autosomes; Mary Lyon was the first to suggest that waystations could be repeat elements such as LINEs (Lyon, 1998). Bioinformatic studies have supported this “repeat hypothesis,” demonstrating that LINEs are generally depleted around escape genes and enriched near subject genes, relative to the rest of the genome (Bailey et al., 2000; Wang et al., 2006). This pattern has held for autosomal genes on X;autosome translocations, suggesting a functional relationship rather than an evolutionary coincidence of both acquisition of repetitive elements and silencing (Bala Tannan et al., 2014; Cotton et al., 2014). LINEs are densest in the oldest strata of the human X (Ross et al., 2005), consistent with the notion of the accumulation of silencing elements over evolutionary time. The mouse, which has fewer escape genes, also has a greater density of LINEs (Ngamphiw et al., 2014). Additionally, pre-existing features of heterochromatin such as polycomb repressive complex marks are strongly associated with genes that become silenced and therefore may also act as waystations (Cotton et al., 2014; Loda et al., 2017).

#### Intrinsic Escape Elements

A number of genes, particularly those with Y gametologs, consistently escape from XCI across multiple species, arguing that their escape is controlled by conserved elements that were present in the ancestral sex chromosomes. Definitive support for intrinsic escape elements was obtained from integrating a BAC transgene containing an escape gene (*Kdm5c*) in four random locations on the mouse Xi (Li and Carrel, 2008). Escape status was recapitulated in that *Kdm5c* always escaped, while the flanking genes present in the BAC remained subject to XCI. The ongoing expression of a primate-specific escape gene (*RPS4X*) that was observed upon integrating a human BAC transgene onto the mouse Xi demonstrated that such elements could be recognized across species (Peeters et al., 2018). This finding validates the use of mouse models to delineate escape elements present in humans or other mammals.

#### Boundary Elements

Boundaries between subject and escape regions on the X have been argued to be established by CTCF sites (Giorgetti et al., 2016), which are well established to create topologically associating domain (TAD) boundaries, marking regions of the genome that are enriched for within-domain interactions (Gómez-Marín et al., 2015). However, since there are over 2000 CTCF sites on the X, there must also be other factors involved in determining what designates escape regions (Ding et al., 2014). The BAC transgene studies mentioned above provided evidence for sequences inhibiting the spread of euchromatin: when the integrated escape region was truncated, spread of escape to flanking genes that are normally subject to XCI was observed (Horvath et al., 2013). In addition to serving as boundaries, CTCF has been reported to associate with promoters of escape genes (Loda et al., 2017), as has the YY1 transcription factor (Chen et al., 2016; Loda et al., 2017).

Boundary elements may be present between the clusters of escape and subject genes in humans; this clustering is an important contributor to the greater number of genes that escape inactivation in humans relative to mice. These escape clusters are notably larger on the human short arm and roughly coincide with TADs (Marks et al., 2015). In mice, only one or two genes within these regions escape XCI (reviewed in Balaton and Brown, 2016). The additional escape genes in these TADs in humans may be due to a “bystander” effect in which adjacent genes also escape because they lack proximal silencing elements or boundaries, features presumably acquired within the mouse TADs. To date, it is unknown whether the clustering patterns in humans or mice is the norm in other mammals.

#### Physical Structure of the X

The physical structure of the Xi likely also influences which genes escape from inactivation. The Xi is comprised of a gene-rich outer rim and repeat-rich internal core (Clemson et al., 2006). Upon silencing, subject genes transition inward, whereas the escape genes remain more peripheral (Chaumeil, 2006). Chromosome conformation capture techniques have allowed determination of the detailed structure of the Xi (Rao et al., 2014; Deng et al., 2015). Both mouse and human have an Xi that forms two major “superdomains,” with the “hinge” region containing the microsatellite *Dxz4/DXZ4* (Deng et al., 2015). *DXZ4* has been shown to bind CTCF exclusively on the Xi (Chadwick, 2008; Horakova et al., 2012). Intriguingly, deletion of the *Dxz4/DXZ4* region leads to loss of Xi superstructure but has a limited effect on transcription from the Xi in either species; paradoxically, the mouse deletion resulted in a tendency for less escape from XCI for variable escape genes (Darrow et al., 2016; Giorgetti et al., 2016). It is possible that the different locations of the hinge between humans and mice might allow greater escape from XCI in humans, where the superdomains are less symmetrical. Additionally, the mouse X chromosome is acrocentric whereas the human X is submetacentric (Figure 1); the need to “traverse” the centric heterochromatin might reduce the strength of the X inactivation signal on the human short arm. Other species have differing centromere locations, including an acrocentric configuration in buffalo (Raudsepp et al., 2012), reinforcing that determining XCI status across eutheria has the potential to provide considerable insight into how genes escape from XCI.

### Approaches for Studying Escape From XCI Across Species

Currently the inactivation status of genes has only been extensively studied in humans and mice. Challenges to studying escape in bulk cell samples across species include random XCI in eutherian females, and the lower expression from the Xi compared to the Xa. We will discuss approaches used in humans and mice, and their potential for studies across eutheria.

The first extensive survey of escape from inactivation in humans utilized somatic cell hybrids (Carrel and Willard, 2005), in which expression from the human Xi could be distinguished from the Xa by separation of the human chromosomes in a mouse background. While this could be used for other eutheria, hybrids bring their own challenges, including loss of XIST localization (Clemson et al., 1998; Hansen et al., 1998) and reactivation of repetitive elements (Ward et al., 2013). Carrel and Willard (2005) also took advantage of expressed single nucleotide polymorphisms (SNPs) to examine allele-specific expression in clonal fibroblast cell lines. More recently, while surveying post-mortem tissue samples [the Genotype-Tissue Expression (GTEx) study], a female with completely non-random inactivation was identified, allowing a similar study of multiple tissues (albeit in a single female) (Tukiainen et al., 2017). In mice, to obtain samples with non-random inactivation, groups have studied extra-embryonic tissues (Mugford et al., 2014), clonal cell lines (Berletch et al., 2015), or engineered *Xist* mutations (Marks et al., 2015). The GTex study also investigated escape using single-cell RNA-seq (scRNA-seq). As each cell has only a single Xa, the presence of biallelic expression indicates escape from XCI, and clonal cell populations are not required. Therefore, this approach could be applied to other species. Currently scRNA-seq is often focused on cell identification rather than deep gene-wide reads that would be informative for XCI status, but new bioinformatic and sequencing approaches are being developed. In mice defined crosses between divergent strains or species with unique polymorphisms allows for greater informativity from allelic expression analyses. Limited heterogeneity in livestock, such as cows, could reduce the informativity of allelic expression assessment of XCI in other species (Couldrey et al., 2017).

In the absence of polymorphisms, comparisons of expression in females relative to males correlates with escape (Tukiainen et al., 2017), although this approach is limited in sensitivity, and hormonally induced gene expression reduces robustness. Extension to aneuploidies can improve sensitivity, but is confounded by impacts of the aneuploidies themselves (discussed further below). By using DNA methylation as a surrogate for inactivation, comparison of males to females has been quite informative, particularly for those genes with CpG-rich promoters that become highly methylated on the Xi (Cotton et al., 2015). Such analyses, while not directly examining expression, need neither clonal populations nor polymorphisms, and thus would be amenable to exploration of additional species. DNA methylation of candidate escape genes provided evidence for escape in moles and cow (Yen et al., 2007). As a caveat, marsupial DNA methylation is not differential at promoters, but rather shows hypomethylation in flanking regions (Waters et al., 2018). Other “marks” of an Xi can also be used to identify XCI status – such as RNA polymerase occupancy (Kucera et al., 2011) or ATAC-seq open chromatin (Qu et al., 2015) – but these have not been used frequently. Assessment of expression by *in situ* hybridization (RNA-FISH) allows the examination of expression from the Xa or Xi within individual nuclei. Biallelic signals (indicative of escape from XCI) were observed in elephant, human, and mouse for several candidate escape genes, although a substantial proportion of cells showed only a single focus of expression (Al Nadaf et al., 2011). This could be a result of the challenge of detecting low levels of expression from the Xi, or a reflection of heterogeneous Xi expression as observed in other studies (e.g., Garieri et al., 2018).

## Implications of Escape From XCI

### Sex-Chromosome Aneuploidy

Sex chromosome aneuplodies occur in approximately 1/500 births and so are among the most common chromosomal abnormalities in humans (Nussbaum et al., 2001). This is presumably due to reduced phenotypic consequences, as all but one X chromosome is silenced, and the Y chromosome has both a reduced number and testis-specialization of genes.

Turner syndrome (TS) results from the absence of a second sex chromosome, although mosaicism and rearranged X chromosomes (such as an isochromosome) are observed in a significant proportion of cases. Strikingly, the vast majority of 45,X conceptuses are spontaneously aborted, which has been argued to reflect a need for some level of mosaicism (Kelly et al., 1992). In spite of this loss, TS is observed in ∼1/2,000 newborn females, leading to infertility, cardiovascular defects, and other neurological and physical abnormalities (Granger et al., 2016). Mice show far less phenotypic consequence of the absence of a second sex chromosome, which may be due to the reduced number of genes that escape inactivation (reviewed in Berletch et al., 2011).

Klinefelter syndrome, usually 47,XXY but sometimes 48,XXXY or 49,XXXXY, occurs in ∼1/650 male births and is accompanied by the inactivation of all but a single X. While infertility and tall stature are common, additional phenotypic consequences vary widely, contributing to an overall increase in morbidity and mortality (Kanakis and Nieschlag, 2018). In mice, the presence of imprinted X inactivation in the extraembryonic tissues causes an extra maternally derived X chromosome to be detrimental to early development (Shao and Takagi, 1990).

There are reports of sex chromosome aneuploidy in other mammals, and by reviewing this literature in comparison to genome assemblies, Raudsepp et al. suggest the viability of XO individuals is strongly correlated with a smaller PAR (Raudsepp et al., 2012). They fail to detect a similar correlation with X chromosome trisomy, implicating the PAR genes in early developmental failure rather than a PAR contribution to non-disjunction. In addition to the rare literature reports of cats with sex-chromosome monosomy, there is also a scientific and lay literature describing male tortoiseshell or calico cats. As black and orange coat color are X-linked alleles, these felines are therefore presumably XX. These have been shown to be both 39,XXY and chimeras (reviewed in Lyons, 2015). Interestingly, the karyotyping of intersex marsupials has suggested that while the Y chromosome is testis-determining, the development of a scrotum or pouch is more likely controlled by a gene(s) on the X chromosome, likely through dosage of a gene that escapes X inactivation (reviewed in Deakin et al., 2012).

### Escape Genes and Disease

Due to the complexity of comparing XX females and XY males, the X chromosome is often excluded from genome-wide association studies, limiting our understanding of its relationship to complex diseases. Conversely, X-linked mutations in hemizygous males are readily detected, and thus a high density of monogenic traits have been assigned despite the relatively low gene density. The X chromosome is subject to unique evolutionary pressures: it spends more time in females, yet the hemizygosity in males allows immediate expression (and therefore positive or negative selection) of new mutations. Thus, the X chromosome has a unique repertoire of genes, and has been described as a “smart and sexy” chromosome due to genes involved in brain and gametogenesis (Graves et al., 2002, reviewed in Disteche, 2016). There are over 141 known X-linked intellectual disability genes, with potential for another 80 currently only mapped as syndromes (Neri et al., 2018). Strikingly, duplications and consequent phenotypes have been observed for the majority of these genes, suggesting that there is considerable dosage sensitivity.

The X chromosome is home to a variety of immune-related genes. Auto-immunity is often more common in females (reviewed in Natri et al., 2019), and is also more prevalent in Klinefelter males than XY males. Furthermore, most cancers are more prevalent in males than females, and immunological differences have been argued to be a contributor to this sex differential (Natri et al., 2019). Additionally, for X-linked genes with tumor-suppression function, only a single mutation in males would result in loss of function. If the gene is subject to XCI, females would also lose function through a single Xa mutation. However, if this gene escapes XCI, then females would be protected through their Xi copy resulting in a lower prevalence in females. This scenario was recently developed into the EXITS hypothesis (escape from X inactivation for tumor suppressors) to explain the male predominance for many cancers (Dunford et al., 2017).

## Concluding Thoughts

The evolution of divergent sex chromosomes led to dosage imbalances – both between the ancestral autosomes and the sex chromosomes, and between males and females. While Ohno proposed up-regulation of the residual X-linked genes (dosage compensation) and silencing of one X chromosome in females (dosage equivalence), studies in the last 50 years have revealed unexpected alternatives. In humans over 25% of genes escape from XCI, and over half of these do not have Y gametologs. Although multiple controlling elements have been suggested, it is currently unknown what DNA sequences, chromatin features, or ultrastructural contributions allow genes to escape from XCI. Identification of these elements will provide insights into heterochromatin, and may inform therapeutics aimed at reactivating X-linked genes, silencing autosomes, or avoiding host silencing of gene therapies. In mice, the use of sex-reversed mice to assess the “four core genotypes” allows researchers to distinguish the impact of the sex chromosomes from sex of the animal, and has revealed chromosomal contributions to many diseases (Burgoyne and Arnold, 2016). Since humans have more escape genes, these contributions could be even larger and thus understanding expression from the sex chromosomes is an important facet in delineating the abundant sex differences in human disease. As the ancestry of X-linked genes led to their ability to escape, exploring dosage compensation and dosage equivalence in more species could provide clues to the balance of features involved.

## Author Contributions

BP and CB each contributed to writing of the review.

## Conflict of Interest

The authors declare that the research was conducted in the absence of any commercial or financial relationships that could be construed as a potential conflict of interest.
